# Quality and Presence of Behaviour Change Techniques in Mobile Apps for the Mediterranean Diet: A Content Analysis of Android Google Play and Apple App Store Apps

**DOI:** 10.3390/nu14061290

**Published:** 2022-03-18

**Authors:** Daniel McAleese, Manolis Linardakis, Angeliki Papadaki

**Affiliations:** 1 Centre for Exercise, Nutrition and Health Sciences, School for Policy Studies, University of Bristol, Bristol BS8 1TZ, UK; daniel.mcaleese@oncology.ox.ac.uk; 2 Department of Oncology, Medical Sciences Division, University of Oxford, Oxford OX3 7DQ, UK; 3 Department of Social Medicine, Faculty of Medicine, University of Crete, 71003 Heraklion, Crete, Greece; linman@med.uoc.gr

**Keywords:** behaviour change techniques, content analysis, Mediterranean diet, mobile apps, mobile health (mHealth), quality, smartphone

## Abstract

Smartphone apps might represent an opportunity to promote adherence to the Mediterranean diet (MedDiet). This study aimed to evaluate the quality of commercially available apps for the MedDiet and the presence of behavioural change techniques (BCTs) used by these apps. A systematic search was conducted on the Apple App and Google Play stores in November 2021. Apps were included if they provided information on the MedDiet or if their objective was to promote a healthy lifestyle through adherence to the MedDiet. Eligible apps were independently evaluated by two reviewers with regard to their quality (engagement, functionality, aesthetics and information quality) using the 5-point Mobile App Rating Scale (MARS; with higher scores indicating higher quality), and the presence of BCTs using an established 26-item BCT taxonomy. Of the 55 analysed apps, 52 (94.5%) were free, 50 (90.9%) provided recipe ideas, 29 (52.7%) provided meal plans, and 22 (40%) provided information on the health benefits of the MedDiet. The overall quality mean MARS score was 2.84 (standard deviation (SD) = 0.42), with functionality being the highest scored MARS domain (mean = 3.58, SD = 0.44) and engagement the lowest (mean = 2.29, SD = 0.61). The average number of BCTs in the analysed apps was 2.3 (SD = 1.4; range: 0–6 per app). The number of BCTs was positively correlated with app information quality (r_rho_ = 0.269, *p* = 0.047), overall MARS score (r_rho_ = 0.267, *p* = 0.049), app subjective quality (r_rho_ = 0.326, *p* = 0.015) and app-specific quality (r_rho_ = 0.351, *p* = 0.009). These findings suggest that currently available apps might provide information on the MedDiet, but the incorporation of more BCTs is warranted to maximise the potential for behaviour change towards the MedDiet.

## 1. Introduction

The Mediterranean diet (MedDiet) is widely acknowledged as a dietary pattern exerting a multitude of health benefits [1]. Characterised by high consumption of olive oil, vegetables, fruits, legumes, nuts and unrefined cereals, low consumption of red meat, meat products and sweets, and moderate consumption of dairy products, fish and alcohol [2], the MedDiet has antioxidant and anti-inflammatory properties that have been proposed to reduce the risk of various non-communicable diseases [3,4]. An umbrella review of meta-analyses of observational and randomised controlled trials [5], as well as meta-analyses of randomised controlled trials [6,7,8,9], found that the MedDiet beneficially affects several metabolic risk factors and is associated with reduced risk of cardiovascular diseases, type 2 diabetes and overall mortality and cancer incidence. Due to these benefits, there is a recognised need to promote this ‘model for healthy eating’ among populations in both Mediterranean and non-Mediterranean settings [2,10,11,12]. It is therefore essential to identify opportunities to promote the MedDiet for public health benefits that would allow wider population reach.

Smartphones and mobile health (mHealth) interventions can access large numbers of individuals at no, or low, cost, and therefore pose an opportunity for implementing programmes of self-managed care [13] and promoting behaviours associated with healthy lifestyles [14,15]. A recent systematic review and meta-analysis of 41 studies, including 27 randomised controlled trials, reported that app-based interventions result in beneficial changes in nutrition behaviours and nutrition-related health outcomes, including obesity indices, blood pressure, and concentrations of blood lipids [16]. The current numbers of smartphone users exceed six billion worldwide, and are projected to exceed 7.5 billion by 2026 [17], whereas in December 2021, the number of mobile apps under the ‘health and fitness’ category available to download from the two major providers were 92,882 in Android’s Google Play [18] and 171,635 in Apple’s App Store [19]. This abundance of health apps makes it increasingly difficult to identify those with the greatest potential to yield positive health benefits to consumers. Indeed, information in commercially available dietary, physical activity and weight management apps is often not evidence-based, and many do not contain theoretical principles that could enhance effectiveness, or are of questionable quality [20,21,22]. The method utilised to assess the quality of an app is also essential, as it has been argued that consumer ratings, which represent one of the primary methods of quantifying the quality of commercial health apps [23], can be misleading and do not necessarily reflect scientific evidence [24]. Instead, validated multi-dimensional measures of quality, which extend beyond user ratings and reviews and focus on functionality, engagement, aesthetics and information quality [24,25], and transparency in reporting, such as providing a list of reviewed apps [23], could allow consumers to make informed choices to fully benefit from mHealth apps. In addition, mHealth apps that contain established behaviour change techniques (BCTs), which are based on theories of behaviour change and aim to provide practical principles to change determinants of behaviour [26,27,28], could facilitate behaviour change [25]. Yet, BCTs are not widely incorporated in commercially available apps for diet, physical activity or weight management [20,21,22,29,30]. In particular, five BCTs (self-monitoring, intention formation, specific goal-setting, review of behavioural goals and feedback on performance) have been linked to effective dietary behaviour change interventions [31] and could be incorporated in dietary mHealth apps to maximise the potential for beneficial effects [22,29,32]. 

To our knowledge, no study to date has evaluated commercially available smartphone apps that provide information on, and potentially promote, the MedDiet, with regard to their quality and incorporation of BCTs. Using a multi-dimensional measure of quality assessment (engagement, functionality, aesthetics and information quality) [24] and an established 26-item taxonomy of BCTs [28], the aim of this study was to conduct a content analysis of mobile apps for the MedDiet (referred to as ‘MedDiet apps’ from here on) available in the Apple App Store and Google Play, and answer the following research questions: (1) What is the quality of MedDiet apps? (2) Do MedDiet apps incorporate BCTs, and if so, which BCTs do they include? (3) What is the relationship between the different domains of app quality and the BCTs incorporated in these apps? As this was an exploratory analysis, with the aim of informing future mHealth intervention development or app development practice, no specific hypotheses were formulated. 

## 2. Materials and Methods

### 2.1. App Identification and Selection

The Apple App Store and Android Google Play Store were searched on 23 November 2021, using the search terms ‘Mediterranean diet’, or ‘Mediterranean lifestyle’ or ‘Mediterranean healthy lifestyle’, or ‘Mediterranean healthy diet’, to identify all available apps related to the MedDiet. The search terms were entered directly into the homepages of the app stores. The search was conducted by the primary researcher (DMcA) using an Apple iPad Air 4th Generation device (version iOS 14.7.1, Apple Inc., Cupertino, CA, USA) to identify apps from the Apple App Store, and a Samsung Galaxy Tab A7 Lite (version Android 11, Samsung Electronics, Suwon, South Korea) to identify apps from the Google Play store. 

The app titles and descriptions were first reviewed against the inclusion and exclusion criteria by the first author, and checked by the third author, to establish relevance to the MedDiet. Apps were included if they provided information on the MedDiet, including, but not limited to, the benefits of the MedDiet, MedDiet recipes, and encouraging adherence to the MedDiet, and if the title and description implied that the app was specifically designed to promote a healthy lifestyle through adherence to the MedDiet. Apps were excluded if they were not available to download in English, if the downloading process was corrupted (i.e., the app was not possible to download), or if the app did not exclusively focus on the MedDiet (e.g., if other diets were also promoted within the app). Unlike previous studies that have examined the content of health apps by selecting apps according to user ratings [20,21], average user ratings were not used as a selection criterion in the current study because their subjective nature does not necessarily provide useful information on app quality [24]. Following the application of the inclusion and exclusion criteria, and discussions to reach consensus on app selection between reviewers, all apps deemed relevant were downloaded, including free apps and those that required a fee to download. If an app was available to download from both stores, it was downloaded from Google Play only.

### 2.2. Data Extraction and Assessment of App Quality and BCTs

The selected apps were assessed for quality and presence of BCTs by two reviewers independently (DMcA and AP) using a standardised Excel form; any disagreements were resolved by discussion between reviewers until consensus was reached. Both reviewers (one nutrition, physical activity and public health Master’s student and one public health nutritionist) had experience in health behaviour change and coding behaviour change techniques, used dietary apps personally and trained in piloting the coding of the MARS domains in three dietary apps not included in this analysis. Before commencing and completing their assessment (which lasted approximately half a day per app), each reviewer spent approximately 15 min on each app to become familiar with its menus and functionality. Each app’s title and developer were recorded, and data were extracted about which platform apps were downloaded from and whether payment was required for download, also recording the price if applicable. 

#### 2.2.1. Assessment of App Quality

App quality was assessed using the Mobile Application Rating Scale (MARS), which has demonstrated excellent inter-rater reliability and excellent internal consistency in earlier work [24]. The MARS scale includes 19 items categorised under four domains: (1) engagement (entertainment, interest, customisation, interactivity and target group; *n* = 5 items); (2) functionality (performance, ease of use, navigation, gestural design; *n* = 4 items); (3) aesthetics (layout, graphics, visual appeal; *n* = 3 items), and; (4) information (accuracy of app description, goals, quality of information, quantity of information, visual information, credibility, evidence base; *n* = 7 items). Each item was assessed using a 5-point scale, and an overall MARS score for each app (the overall quality mean score) was calculated by summing the mean scores for each of the four domains, and dividing by four. App subjective quality (intention to recommend the app to others, perceived number of times the app will be used in the next 12 months, intention to pay for the app, overall star rating of the app; *n* = 4 items), using similar 5-point scales, was also assessed and a subjective quality mean score calculated. Finally, app-specific quality, which aims to evaluate the perceived impact of the use of an app on awareness (likelihood of increasing awareness of the importance of addressing the MedDiet), knowledge (likelihood of increasing knowledge of the MedDiet), attitudes (likelihood of changing attitudes toward adhering to the MedDiet), intentions to change (likelihood of increasing motivation to address the MedDiet), help seeking (likelihood of encouraging further help seeking for the MedDiet) and behaviour change (likelihood of increasing adherence to the MedDiet) (*n* = 6 items), was assessed using 5-point scales (from ‘strongly disagree’ to ‘strongly agree’) [24]. Overall, MARS scores ranged from zero to five, with higher scores indicating higher app quality. The full MARS can be found as supplementary material in the paper by Stoyanov et al. [24].

#### 2.2.2. Assessment of Presence of BCTs

The presence or absence of BCTs in the included apps was assessed using the taxonomy of BCTs developed by Abraham and Michie [28]. This taxonomy consists of 26 BCTs, mapped onto established theoretical frameworks, which could be utilised to evaluate the effectiveness of behaviour change interventions, and has been utilised for the evaluation of the presence of BCTs in similar studies [21,22,33,34]. The 26 BCTs assessed (provides instruction, provides information on behaviour-health risks, provides information on consequences, prompts intention formation, models or demonstrates behaviour, prompts practice, prompts self-monitoring, prompts barrier identification, time management, motivational interviewing, relapse prevention, prompts identification as role model, teaches to use environmental prompts or cues, provides contingent rewards, provides feedback on performance, prompts review of behavioural goals, prompts specific goal setting, sets graded tasks, provides general encouragement, provides information on others’ approval, agrees on behavioural contract, use of follow-up prompts, provides opportunities for social comparison, plans social support or social change, prompts self-talk, and stress management) were entered into a checklist and their presence or absence from each app was assessed (0: absent, 1: present).

### 2.3. Data Analysis

Data were analysed using SPSS software (IBM SPSS Statistics for Windows, Version 26.0. IBM Corp., Armonk, NY, USA). Inter-rater reliability regarding the MARS scores and the number of BCTs present in the analysed apps was assessed using two-way, mixed effects intraclass correlation coefficients (ICC). ICCs of <0.4 would prompt the need for the reassessment of apps, whereas ICCs of >0.6 were considered to indicate good agreement, and those of >0.7 indicated excellent agreement between raters [35]. Internal consistency of the MARS scale and BCTs were assessed using the Cronbach’s α (alpha) and the Kuder–Richardson index (KR-20), respectively. App quality and BCT data were presented descriptively, using means and standard deviations (SD) for continuous variables, and frequencies and percentages for categorical variables. Differences in MARS scores and number of present BCTs between the platforms (Apple App Store vs. Google Play) were assessed, as part of an exploratory analysis, using Mann–Whitney tests, whereas differences in MARS scores according to the number of present BCTs (0–1, 2 and ≥3) were assessed using the Kruskal–Wallis test. The associations between the different domains of app quality and number of BCTs present in the assessed apps were examined using Spearman’s rank correlation coefficients.

## 3. Results

### 3.1. Identification of Apps

**Figure 1:**
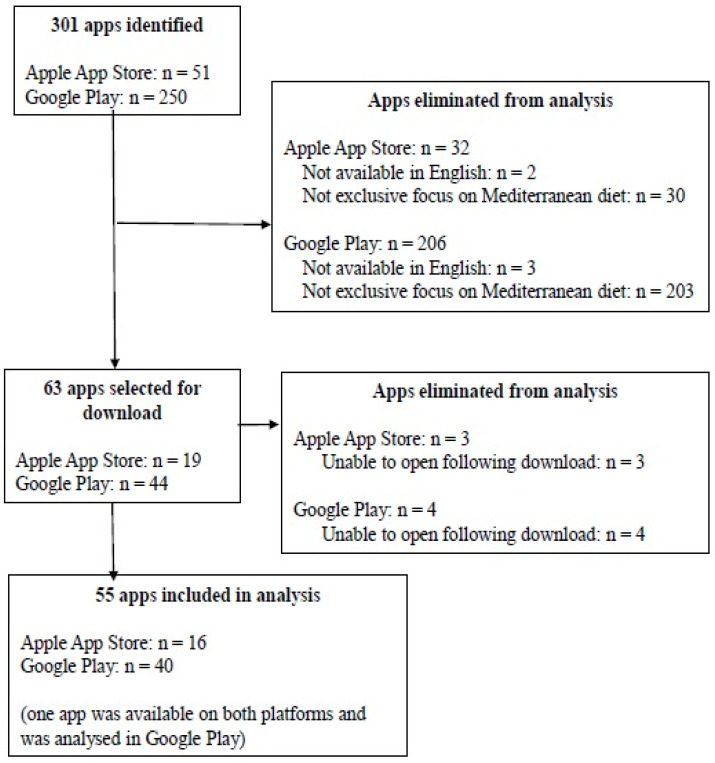
Flow diagram of app search and selection.

Figure 1 illustrates the flow diagram of the app search and selection process. Applying the search terms resulted in the identification of 301 apps (51 in the Apple App Store and 250 in Google Play). Screening against the inclusion and exclusion criteria led to 55 unique apps being included in the analysis (16 from the Apple App Store and 40 from Google Play). One app (PEP: Mediterranean Diet—Food tracker and recipes byBlack Bears) was available from both platforms and was analysed in Google Play (Figure 1). A full list of all the apps analysed in the current study, with information on their developer, platform, cost, mean MARS overall, and domain, scores and presence of BCTs, can be found in Supplementary Materials, Table S1.

### 3.2. App Characteristics and Features

The majority of the apps were free to download (52 apps, 94.5%). The three apps that required payment (on a one-time basis) were available to download from the Apple App Store only, with prices ranging from GBP 0.99 to GBP 1.79 and an average price of GBP 1.52 (USD 2.05) (Supplementary Materials, Table S1). The most prevalent features of the analysed apps were recipe ideas (50 apps, 90.9%) and the provision of meal plans (29 apps, 52.7%). The proportion of apps with recipe ideas or meal plans was higher in apps from the Apple App Store, compared to Google Play (15 apps, 100% vs. 34 app, 85%). In addition, 22 apps (40% of total apps; six (40% of Apple apps) from the Apple App Store and 16 (40% of Google apps) from Google Play) exclusively contained recipe ideas or meal plans, offering no further features or information on the MedDiet.

Another feature of the analysed apps was the provision of information detailing the benefits of adhering to the MedDiet on health outcomes (22 apps, 40.0%). Seventeen apps (30.9% of total apps; six (40% of Apple apps) from the Apple App Store and 11 (27.5% of Google apps) from Google Play) referred to information from the scientific literature when describing potential benefits of the MedDiet. Of these, only three apps (5.5% of the total sample) provided actual citations, and none provided complete references. Additionally, 10 of these 17 apps provided information on health benefits other than weight loss or reduction to the risk of heart disease. Other, more infrequently observed, features of the analysed apps were the presence of a calorie counting feature (7 apps, 12.7%); the presence of a body mass index calculator (5 apps, 9.1%; one from the Apple App Store), and the presence of a food checklist, aiming to educate users on the foods characteristic of the MedDiet (5 apps, 9.1%).

### 3.3. App Quality

Table 1 demonstrates the overall MARS score and the scores for each MARS domain of the assessed apps. The inter-rater reliability for the assessment of app quality was excellent, with an ICC of 0.97 (95% confidence intervals (CI) 0.95–0.98) for the overall MARS score, 0.92 (95% CI 0.86–0.95) for engagement, 0.93 (95% CI 0.89–0.96) for functionality, 0.96 (95% CI 0.93–0.98) for aesthetics, 0.95 (95% CI 0.91–0.97) for information, 0.91 (95% CI 0.80–0.95) for subjective app quality and 0.89 (95% CI 0.81–0.94) for app-specific quality. The internal consistency for the overall score and sub-scores of MARS was deemed excellent (Cronbach’s α > 0.70) (Table 1).

The mean overall MARS score was 2.84 out of 5, ranging from 2.0 to 3.8, indicating moderate overall quality. Functionality was the MARS domain that scored the highest, followed by aesthetics, information and engagement; there was strong evidence to suggest that app functionality was assessed as being of higher quality than app engagement (3.58 vs. 2.29, *p* < 0.001). The mean subjective quality score was 1.69, suggesting low quality of the assessed apps, and the mean app-specific quality score was 2.86, suggesting moderate quality of the assessed apps in changing awareness, knowledge, attitudes, intentions, help seeking and increasing behaviour change (Table 1).

The overall app quality score did not differ according to the platform that the apps were downloaded from. However, functionality was assessed as being of higher quality when apps were available to download from the Apple App Store, compared to Google Play (3.78 vs. 3.51, *p* = 0.027) (Table 2).

### 3.4. Presence of BCTs

**Figure 2:**
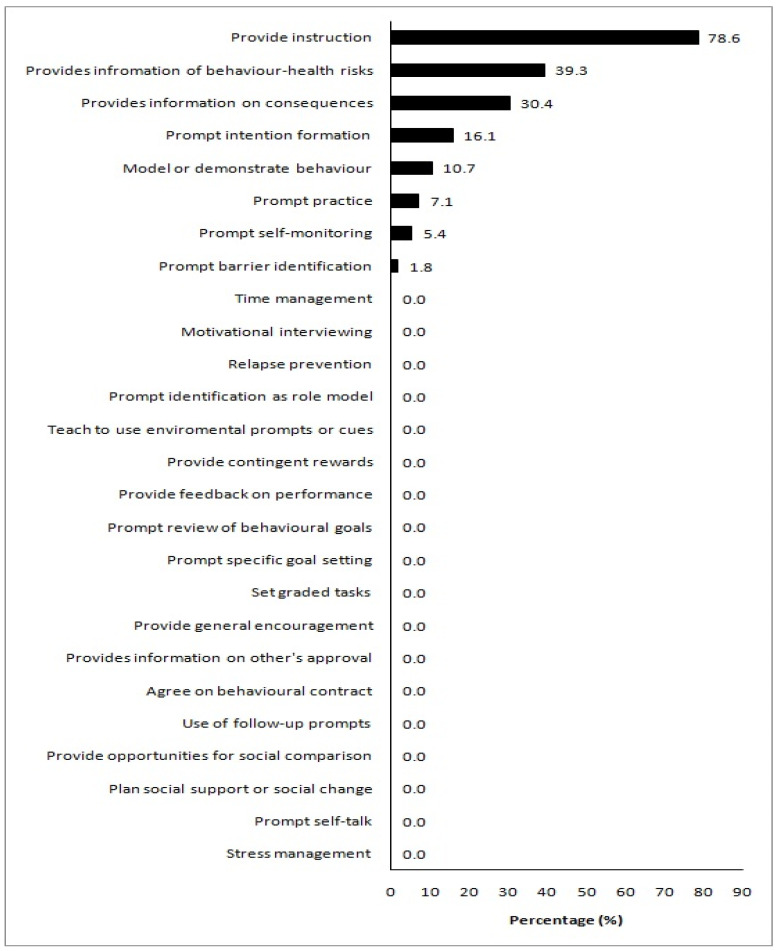
Presence of individual behaviour change techniques in the analysed apps. Values on the *x*-axis denote the proportion of apps a behaviour change technique was present in.

The inter-rater reliability for the assessment of the number of BCTs present in the analysed apps was excellent, with an ICC of 0.94 (95% CI 0.90–0.97). The internal consistency for the assessment of BCTs was also deemed excellent (Kuder-Richardson > 0.70) (Table 1). The mean number of BCTs present in the apps analysed in the current study was 2.3 out of a possible 26, ranging from 0 to 6, indicating a low presence of BCTs in the assessed apps. One app did not contain any BCTs, and only three apps contained the maximum observed number of six BCTs (Supplementary Materials, Table S1). Figure 2 illustrates the proportion of analysed apps assessed as having a BCT present. The most frequently identified BCT was ‘provide instruction’ (44 apps, 78.6%), usually through the provision of recipes and meal plans. ‘Provide information on behaviour health risk’ (22 apps, 39.3%), and ‘provide information on consequences’ (17 apps, 30.4%) were the next most prevalent BCTs. Eighteen BCTs were not present in any of the assessed apps (‘time management’, ‘motivational interviewing’, ‘relapse prevention’, ‘prompt identification as role model’, ‘teach to use environmental prompts or cues’, ‘provide contingent rewards’, ‘provide feedback on performance’, ‘prompt review of behavioural goals’, ‘prompt specific goal setting’, ‘set graded tasks’, ‘provide general encouragement’, ‘provide information on others’ approval’, ‘agree on behavioural contract’, ‘use of follow-up prompts’, ‘provide opportunities for social comparison’, ‘plan social support’, ‘prompt self-talk’, and ‘stress management’) (Figure 2).

**Figure 3:**
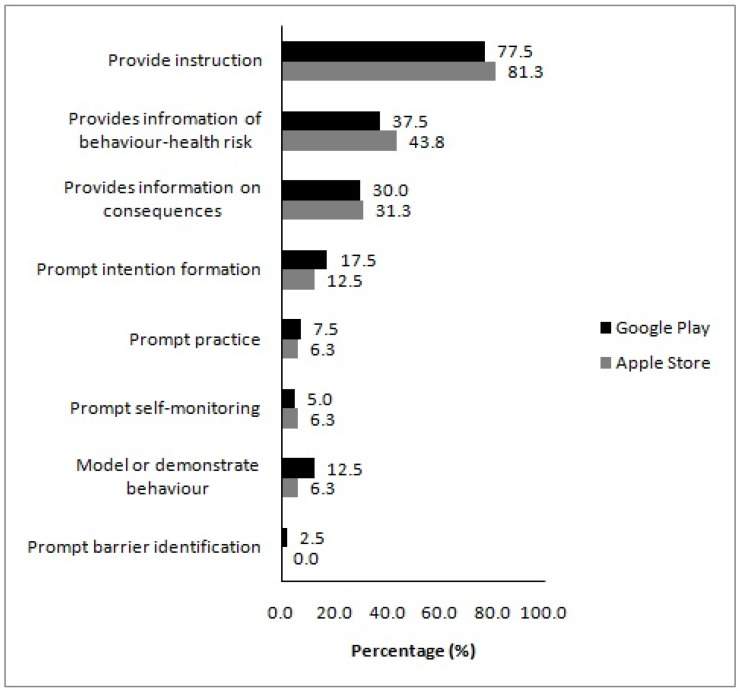
Presence of individual behaviour change techniques in the analysed apps, according to platform. Values on the *x*-axis denote the proportion of apps a behaviour change technique was present in.

The mean number of present BCTs in the assessed apps did not differ by platform (Table 2). Presence of individual BCTs in apps from different platforms followed a similar pattern with the overall analysis, with ‘provide instruction’ being the most prevalent BCT in apps downloaded from both the Apple App Store and Google Play (Figure 3). Overall, seven BCTs were present in at least one app downloaded from the Apple AppStore, compared to eight from Google Play.

### 3.5. Relationship between App Quality and the Presence of BCTs

The number of identified BCTs in the analysed apps was positively correlated with the app mean information quality score (r_rho_ = 0.269, *p* = 0.047), the overall mean MARS score (r_rho_ = 0.267, *p* = 0.049), the app mean subjective quality score (r_rho_ = 0.326, *p* = 0.015), and the app-specific quality score (r_rho_ = 0.351, *p* = 0.009) (Table 3).

Apps that were assessed as having ≥3 BCTs scored higher on the quality domains of engagement, aesthetics, information, subjective quality and app-specific quality, as well as overall app quality, compared to apps assessed as having none or one BCT present (Table 4).

## 4. Discussion

### 4.1. Main Findings

This study aimed to assess the quality and presence of BCTs in commercially available apps which focus on the MedDiet. The most prominent features of the analysed apps were the provision of recipe ideas, meal plans and information on the health benefits of the MedDiet. The apps analysed were overall of moderate quality and scored the highest for functionality and the lowest for engagement. A low incorporation of BCTs was observed, with the most prevalent BCTs involving the provision of instruction and information on behaviour-related health risks and consequences. Although associations were weak, the number of BCTs was positively associated with the overall quality of the apps, while no notable differences in quality or the presence of BCTs were observed between app platforms. These findings have several important implications for the design of future mHealth applications aiming to promote adherence to the MedDiet.

### 4.2. App Features

The vast majority of apps (91%) provided MedDiet recipes, which is a particularly important feature when considering promoting higher adherence to the MedDiet, via an app, to people residing in non-Mediterranean countries, who might not be familiar with this dietary pattern. This was confirmed by an earlier work evaluating a healthy eating website promoting the MedDiet among university employees in Scotland, where recipes represented the most visited website section [36]. Recipes were also perceived to be the most desirable feature of a web-based application aiming to promote the MedDiet among employees in South-West England [37]. Apart from recipes and the provision of information on meal plans and MedDiet health benefits, however, interactive features in the analysed MedDiet apps were sparse; very few apps provided interactivity, which was limited to calorie-counting and body mass index calculators. An earlier content analysis of weight management apps showed that apps providing interactive features, such as tracking behaviour, scored higher in the quality domain of engagement [20]. This could explain the low engagement score that apps received in the current study. Interactive features that provide tailored practical advice have been perceived as being more helpful in changing dietary behaviour and engaging with web-based applications, compared with features that rely on information provision [36], while desirable interactive features of MedDiet apps include personal trackers for reviewing goals, the provision of interactive feedback, discussion fora and online chats with health professionals [37]. As the increased incorporation of interactive features is linked to the effectiveness of [38,39,40], and engagement with [41], health-related web-based applications, interactivity should be an essential element when developing future quality apps to promote the MedDiet, while retaining the feature of recipe provision.

### 4.3. App Quality

The overall quality of the analysed apps was deemed to be moderate, with a mean MARS score of 2.84. This score was slightly lower to the overall quality score reported by earlier studies using MARS to assess apps promoting physical activity (3.88) [21], weight management (3.2) [20] and diet, physical activity and sedentary behaviour in children and adolescents (3.6) [34]. It is noteworthy that these studies analysed the most popular apps in their respective field [20,21], or apps with high user ratings [34], measures which, in practice, are often used as a proxy for app quality [23]. Despite the argument that high user ratings do not necessarily reflect scientific evidence or quality itself [24], it might still be that high ratings are due to some quality domains being more prevalent in these apps. Therefore, the slightly lower overall quality score in the current study might be due to our selection criteria, as we did not exclude apps with low popularity or user ratings.

In addition, the subjective quality of the analysed MedDiet apps was deemed low, with an average score of 1.69. Although subjective quality is a domain of MARS [24], no previous study, to our knowledge, has reported scores for this domain, potentially due to subjective bias introduced by researchers’ previously held opinions of what constitutes a high-quality app. However, establishing an app’s overall quality score by only considering engagement, functionality, aesthetics and information may not represent a true measure of quality, if mHealth users still do not subjectively perceive that an app is of sufficiently high quality to recommend to others, use in the future and pay to download (i.e., the items assessed as part of the subjective quality domain of MARS). Therefore, the overall quality score, combined with the low subjective quality score, suggests that currently, commercially available MedDiet apps are likely of low quality, and that developers of apps focusing on the MedDiet should continually reassess those apps’ features to enhance both overall, and subjective quality.

Functionality was the MARS domain that scored the highest, followed by aesthetics. This finding is in agreement with earlier studies assessing the quality of health-related apps using MARS [20,21,34]. Functionality refers to the overall performance of app functions, the app’s ease of use and navigation and gestural design [24]. Therefore, an app could be assessed as being highly functional and receive a high score for aesthetics, independent of the features it contains or the information it provides. For example, even though recipes were the most prevalent feature in the analysed MedDiet apps, while interactive features were sparse, apps still received high scores for functionality and aesthetics if this information was easy to use and navigate, had an attractive layout, and was visually appealing. Apps that are functional and easy to use are increasingly preferred by mHealth users [37,42,43], while an attractive appearance and visual aspects have also been suggested to be desirable features of web-based applications promoting the MedDiet [36,37]. Therefore, such features should continue to be incorporated, and further enhanced, in future MedDiet apps.

Information quality and engagement were the MARS domains that scored the lowest, confirming the results of earlier studies utilising MARS [20,21,34]. As discussed earlier, the low engagement score can be explained by the limited interactive features in the analysed apps. As app engagement is linked to effectiveness of digital interventions for behaviour change [44] and dietary behaviour change [45], more research is needed to understand the factors that would enhance effective engagement with MedDiet apps among users. With regard to the low scores received for information quality, Simões et al. highlighted that app developers might be more concerned with whether an app is functional and aesthetic than whether it provides high quality, evidence-based information [21]. In their study assessing the quality of popular physical activity apps, only one of the 51 apps reviewed mentioned the involvement of health professionals in developing the app’s content. A lack of evidence-based content was also observed in a study of popular weight management apps [20]. In the current study, we found that 31% of analysed apps referred to the scientific literature when providing information on the benefits of the MedDiet, but only 6% of the apps provided actual citations to scientific studies. Earlier work identified that information quality is an expected feature of web-based applications that would help people follow the MedDiet [37], while source credibility and the provision of scientific evidence to support information content are deemed important to assess an application’s trustworthiness [36,46]. These findings indicate that developers of MedDiet apps should invest in the provision of evidence-based, clearly signposted scientific information, in order to enhance the apps’ quality.

### 4.4. Presence of BCTs

Earlier studies found a low overall presence of BCTs in popular apps targeting physical activity (*n* = 6) [21]; diet, physical activity and sedentary behaviour (*n* = 6) [34], and; weight management (*n* = 10) [20], top-ranked apps for physical activity (*n* = 4.2) [29], and; physical activity and diet (*n* = 8.1) [22], and apps for physical activity (irrespective of user ratings, *n* = 5 BCTs) [33]. Four of these earlier studies [21,22,33,34] utilised the same, 26-item, taxonomy of behaviour change [28] to evaluate the presence of BCTs, therefore allowing more direct comparisons with our findings. The current study reported an even lower number of BCTs in the MedDiet apps analysed; this ranged from 0 to 6, with a mean of 2.3 BCTs per app. An earlier study assessing whether the content of 58 dietary apps, identified from the Apple App Store Health and Fitness category, was guided by theories of behaviour change also found that most apps were not informed by theory [47]. It should be noted that comparisons of the presence of BCTs with previous studies is hindered by the use of different app selection criteria, different targeting behaviours and different platforms used to identify apps. Nevertheless, these findings highlight the scarcity of BCTs in commercially available health-related apps. This agrees with findings from a systematic review of studies evaluating apps focusing on one or more health conditions [25], and the systematic review by Villinger et al. of 41 studies examining the effect of app-based interventions on nutrition behaviours and nutrition-related health outcomes, which found that out of a possible 93 BCTs, the average number of BCTs addressed across these studies was 6.9 (range: 2–11) [16]. As the current study showed that the number of BCTs is positively correlated with app quality, this further supports the need for MedDiet app developers to not only ‘provide instruction’ (the most prevalent identified BCT in the current study) but also incorporate appropriate combinations of BCTs in MedDiet apps to improve app quality and help users towards following the MedDiet.

It is noteworthy that of the five BCTs that have been linked to greater effectiveness of dietary and physical activity interventions (‘prompt intention formation’, ‘prompt self-monitoring’, ‘provide feedback on performance’, ‘prompt review of behavioural goals’, and ‘prompt specific goal setting’) [31], only intention formation and self-monitoring were identified in the MedDiet apps analysed, albeit only in 16% and 5% of the apps, respectively. Earlier work has suggested that goal setting, monitoring behaviour and provision of feedback on behaviour are important components of dietary behaviour change apps targeting weight management, as these BCTs help app users to improve their diet [48], and that apps that incorporate BCTs to enhance motivation and self-efficacy, as well as goal-setting tools, might be useful for dietary behaviour change [45]. A tailored approach to goal-setting, reviewing goal progress, and receiving expert feedback were also deemed important to help users of web-based applications to follow a MedDiet [37]. Furthermore, the review of app-based interventions for dietary behaviours by Villingeret al. highlighted that ‘goals/planning’ and ‘feedback/monitoring’ were two of the most prevalent BCT clusters utilised in interventions [16]. Nevertheless, this review analysed studies which utilised a total of 30 smartphone apps, 15 of which were pre-existing commercial apps, and it might be that apps that had been developed for research purposes had a high prevalence of these BCTs. These findings suggest that commercially available MedDiet apps would be unlikely to promote behaviour change towards adherence to the MedDiet, which is supported by the moderate score that these apps received for app-specific quality. This might be due to the high proportion (40%) of apps that exclusively contained recipe ideas or meal plans, offering no further features, such as tracking adherence to the MedDiet, monitoring progress, or setting goals to achieve adherence to this dietary pattern. However, more research is needed to determine the optimal number of BCTs and establish the effectiveness of these apps towards behaviour change. In addition, future research should examine whether these five BCTs are also associated with the greatest effects in mHealth diet and physical activity interventions.

Engaging social support has also been deemed an important feature of web-based interventions that would help individuals to adhere to the MedDiet [37]. Social support was found to be one of the most prevalent BCT clusters in a review of the effectiveness of app interventions on dietary behaviours [16]. An earlier analysis of physical activity and nutrition apps found that approximately 55% of the apps analysed incorporated the BCTs ‘plan social support’ and ‘provide opportunities for social comparison’ [22]. Similarly, a review of dietary, physical activity and sedentary behaviour apps for children and adolescents found that 40% of the apps analysed included ‘opportunities for social comparison’ [34]. However, the prevalence of BCTs in both these studies was presented for the combination of dietary and physical activity apps, which hinders our ability to establish the extent to which these BCTs are common in dietary apps. It might be that these BCTs are more prevalent in physical activity apps, particularly if these apps can be linked to external devices (such as wearable trackers) that provide opportunities for social comparisons of performance of the behaviour, and, via this route, opportunities for social support.

The low number of paid apps in the current analysis did not allow for a meaningful comparison of quality or presence of BCTs according to whether an app required payment to download or not. Apps that required payment were assessed as having higher quality in an earlier study focusing on mobile apps for the Dietary Approaches to Stop Hypertension (DASH) diet [49]. With regard to the presence of BCTs, evidence from earlier studies is inconsistent. For example, a review of commercial weight loss apps found that apps requiring payment were no more likely, compared to free apps, to include behavioural strategies for weight loss [30], while a review of physical activity apps also found no difference in the number of BCTs between paid and free apps [33]. In contrast, a review of physical activity and dietary apps from New Zealand platforms identified slightly more BCTs in paid, compared to free, apps (9.7 vs. 6.6) [22]. Similar to the review of Middelweerd et al. [33], no differences in the number of BCTs were observed across platforms. More research is needed to examine the role of payment requirements, as well as the platform an app is downloaded from, in app quality and implications for behaviour change.

### 4.5. Strengths and Limitations

To our knowledge, this is the first study to assess the quality and presence of BCTs in commercially available apps focusing on the MedDiet, adding importantly to the existing evidence base of reviews of apps tackling other health behaviours. We searched apps from the two major providers and used a comprehensive search strategy to select the analysed apps. Unlike other studies [20,21,29,30,34], we analysed all MedDiet apps that met our selection criteria, irrespective of user ratings or popularity, thereby providing a comprehensive evaluation of all available apps for the MedDiet. We also used the validated MARS, instead of user ratings, to assess these apps’ quality, and an established taxonomy of BCTs to assess the use of behaviour change components. Finally, we have provided a list of the analysed apps for transparency purposes; this method has been suggested as good practice for quality reviews of health-related apps, as it might aid consumers and health professionals alike to make informed choices, but might also motivate app developers to improve their app’s content [23].

Nevertheless, several limitations that hinder the external validity of the study should be acknowledged. Assessment of the apps often took place in a relatively short timeframe, which might have led to some app features and BCTs being missed, such as those BCTs that require prolonged use (e.g., follow-up prompts). Additionally, the assessment might have produced different results if it had been performed by app users, instead of researchers, or by members of the general public with characteristics (e.g., with regard to age or sex) different to the assessors who conducted the current evaluation. These issues were overcome to a degree by having two researchers evaluating the apps independently, with excellent degree of inter-rater reliability, thereby reducing any potential sources of bias. However, the optimal timeframe needed to assess an app’s quality and the presence of BCTs should be further explored in future studies. In addition, apps were assessed for eligibility by screening their titles and descriptions against the inclusion and exclusion criteria, and therefore apps that might not use our search terms in their title and/or description might have been overlooked. Nevertheless, we followed a comprehensive selection process, similar to earlier studies [20,21,22,34], and we believe that it is unlikely for an app that focuses on the MedDiet (our main inclusion criterion) to not declare this in the title and/or description. Further, due to the low number of apps that required payment, it was not deemed possible to compare the quality and presence of BCTs between free and paid apps. In addition, the current study was only able to access MedDiet apps from the Google Play and Apple App Store platforms in the UK, and it is possible that MedDiet apps available from the respective stores in other countries have different features, quality and incorporated BCTs.

## 5. Conclusions

The present study evaluated, for the first time, the quality and presence of BCTs in currently available commercial apps for the MedDiet. Considering the MedDiet’s renowned health benefits and the potential for mHealth to promote adherence to this dietary pattern, our findings are important for consumers who are considering utilising MedDiet apps to adopt this dietary pattern and health professionals who are considering utilising mHealth to facilitate dietary behaviour change among their clients. Our findings should also prove useful to MedDiet app developers during future updates of the apps analysed. However, the analysed apps’ quality was deemed to be moderate to low, and apps incorporated a low number of BCTs. Further work is essential to improve the available MedDiet apps’ engagement and information quality, as well as to identify the appropriate combination of BCTs that would likely affect dietary behaviour change, before establishing these apps’ effectiveness in helping to increase adherence to the MedDiet.

## Figures and Tables

**Table 1:** App quality and behaviour change technique assessment scores.

	Mean	SD	Median	Min	Max	Cronbach’s α
Engagement (5 items)	2.29	0.61	2.00	1.60	3.60	0.850
Functionality (4 items)	3.58	0.44	3.75	1.75	4.25	0.761
Aesthetics (3 items)	2.83	0.59	3.00	1.33	4.00	0.831
Information (7 items)	2.67	0.54	2.67	1.00	3.80	0.710
App Quality (overall mean)	2.84	0.42	2.81	1.98	3.78	0.864
App subjective quality (4 items)	1.69	0.42	1.50	1.25	2.75	0.735
App-specific quality (6 items)	2.86	0.61	2.83	2.00	4.00	0.894
Number of BCTs (26 items)	2.3	1.4	2.0	0	6	0.752 ^a^

^a^ Kuder–Richardson index (KR-20). Responses of all MARS domains ranged from 1 to 5. Higher scores indicated a higher degree of app quality. Kruskal–Wallis test between the four main domains of MARS: *p*-value < 0.001. BCTs: behaviour change techniques; SD: standard deviation.

**Table 2:** App quality and behaviour change technique assessment scores according to app platform.

	App Platform				
	Apple App Store(*n* = 15)		Google Play (*n* = 40)		
	Mean	SD	Mean	SD	*p*-Value
Engagement	2.23	0.54	2.32	0.64	0.863
Functionality	3.78	0.27	3.51	0.47	0.027
Aesthetics	2.96	0.64	2.78	0.57	0.454
Information	2.50	0.59	2.74	0.51	0.160
App Quality (overall mean)	2.87	0.41	2.84	0.43	0.770
App subjective quality	1.63	0.46	1.71	0.41	0.394
App-specific quality	2.90	0.59	2.85	0.62	0.683
Number of BCTs	1.9	1.4	2.4	1.4	0.084

Responses of all items in MARS ranged from 1 to 5. Higher scores indicated a higher degree of app quality. Differences in scores and numbers of BCTs between platforms were assessed using Mann–Whitney tests. BCTs: behaviour change techniques; SD: standard deviation.

**Table 3:** Correlations between MARS scores and number of behaviour change techniques in the analysed apps.

MARS Domain	Spearman Rank Correlation Coefficients	*p*-Value
Engagement	0.252	0.064
Functionality	0.121	0.378
Aesthetics	0.225	0.099
Information	0.269	0.047
App quality (overall mean)	0.267	0.049
Apple App Store app quality (overall mean)	0.251	0.368
Google Play app quality (overall mean)	0.295	0.065
App subjective quality	0.326	0.015
App-specific quality	0.351	0.009

Responses of all items in MARS ranged from 1 to 5. Higher scores indicated a higher degree of app quality. Correlations between the mean MARS scores and the number of BCTs were assessed using Spearman rank correlation. MARS: Mobile Application Rating Scale.

**Table 4:** App quality assessment scores according to presence of behaviour change techniques.

	Behavioural Change Techniques			
	0 or 1 (*n* = 16)	2 (*n* = 24)	≥3 (*n* = 15)	
MARS Domain	Mean (SD)			*p*-Value
Engagement	2.23 (0.54)	2.13 (0.56)	2.61 (0.66)	0.046
Functionality	3.58 (0.55)	3.49 (0.38)	3.73 (0.36)	0.158
Aesthetics	2.81 (0.58)	2.64 (0.53)	3.16 (0.58)	0.047
Information	2.63 (0.45)	2.48 (0.48)	3.03 (0.56)	0.017
App Quality (overall mean)	2.81 (0.37)	2.69 (0.38)	3.13 (0.42)	0.012
App subjective quality	1.59 (0.38)	1.58 (0.37)	1.97 (0.46)	0.021
App-specific quality	2.75 (0.38)	2.64 (0.58)	3.34 (0.61)	0.004

Responses of all items in MARS ranged from 1 to 5. Higher scores indicated a higher degree of app quality. Differences in scores according to the presence of BCTs were assessed using the Kruskal–Wallis test. MARS: Mobile Application Rating Scale; SD: standard deviation.

## Data Availability

The data presented in this study are available in Supplementary Materials, Table S1. Other data that support the findings of this study are available from the corresponding author upon reasonable request.

## Supplementary Materials

The following supporting information can be downloaded at: https://www.mdpi.com/article/10.3390/nu14061290/s1, Table S1: Characteristics, MARS scores and number of behaviour change techniques in the analysed apps.
