# Supplementary material for: Quality and Presence of Behaviour Change Techniques in Mobile Apps for the Mediterranean Diet: A Content Analysis of Android Google Play and Apple App Store Apps
Source: Nutrients. 2022 Mar 18;14(6):1290. doi: 10.3390/nu14061290 (PMC8950036; doi:10.3390/nu14061290)
Supplement: Supplementary file 1 [file nutrients-14-01290-s001.zip › nutrients-1619470-supplementary.pdf]

Table S1. Characteristics, MARS scores and number of behaviour change techniques in the analysed apps

Platform	App Name	Developer	Price (£)	Engagement	Functionality	Aesthetics	Information	Quality mean score	Subjective quality	App-specific quality	Number of BCTs
**Apple Store**	MD- Lower Heart Disease Risk with Nutrition Tips	Randel Smith	Free	2.00	3.75	2.33	1.00	2.27	1.25	2.83	2
	MD Recipes Cookbook	Entertainment Ventures LLC	Free	2.20	3.75	3.00	2.50	2.86	1.50	3.33	4
	MD	Mark Patrick Media	1.79	2.60	4.00	3.00	2.75	3.09	2.00	2.17	2
	MD Recipes	Yan Lee	1.79	2.60	4.00	2.67	2.75	3.00	1.50	3.00	1
	My MD Tracker	Prestige Worldwide Apps LLC	Free	3.60	4.00	3.67	3.80	3.77	2.75	2.50	6
	MD & Meal Plan	Realized LLC	Free	2.00	3.75	3.33	2.25	2.83	1.25	3.00	1
	MD & Recipes	Content Arcade Dubai Ltd.	Free	1.60	3.75	2.67	2.25	2.57	1.25	2.67	2
	MD: recipes, meal plans and food list	Mikhail Davydov	0.99	2.20	3.50	3.00	2.25	2.74	1.25	2.83	1
	MD Meal Plan	Smart Query	Free	1.60	3.50	2.00	2.60	2.43	1.25	2.50	1
	MD Plan: Low Carb Diet	Diego Correa Bonini	Free	2.00	3.50	2.33	2.40	2.56	1.50	3.17	1
	MD Meal Plan	Heyleen Pulgar	Free	2.20	4.00	3.33	3.20	3.18	2.00	2.83	1
	MD healthy app	Moran Navon	Free	2.80	3.75	3.00	2.80	3.09	1.50	2.67	2
	MD + Meal Plan	Nikita Gnedin	Free	1.60	4.00	4.00	2.25	2.96	2.00	2.83	3
	MD Plan	Luis Alberto Hurtado	Free	2.60	4.25	4.00	2.50	3.34	2.25	2.00	1
	Mediterranean food	Luis Borrero	Free	1.80	3.25	2.00	2.25	2.33	1.25	2.33	1
**Google Play**	MD Plan	Chelin Apps	Free	3.40	4.25	3.00	3.20	3.46	2.50	3.83	6
	Medidiet	Medidiet team	Free	3.00	3.25	3.67	2.00	2.98	1.25	3.33	4
	MD Plan	Chiquito Apps	Free	2.40	4.00	3.33	3.20	3.23	2.00	2.00	5

Table S1 (continued)

Platform	App Name	Developer	Price (£)	Engagement	Functionality	Aesthetics	Information	Quality mean score	Subjective quality	App-specific quality	Number of BCTs
**Google Play**	MD & Meal Plan	Healthy. Happy. Smart	Free, but requires payment after a week of use	3.20	4.00	4.00	3.75	3.74	2.50	2.00	3
	1500+ MD Recipes Offline	Edutainment Ventures	Free	2.00	3.75	2.67	2.80	2.80	1.50	4.00	0
	MD Recipes: Mediterranean Recipes	Supine Lab	Free	2.20	4.00	3.33	3.00	3.13	2.00	2.50	1
	MD Free Plan	FYTD	Free	2.40	3.75	3.33	3.20	3.17	2.25	3.17	3
	The MD Beginner's Guide	App4Life	Free	2.00	4.00	3.00	3.20	3.05	1.50	2.50	2
	MyMedi: MD Tracker & Meal Plan	Prestige Worldwide Apps	Free, but full features require payment	3.60	4.25	3.67	3.60	3.78	2.50	3.00	6
	MD Receipes for free app offline	Cooking Recipes Book	Free	2.00	3.50	2.67	3.25	2.85	1.50	2.83	1
	MD Weight Loss Plan	Best Top Daily Guide and Info	Free	2.00	3.50	3.00	2.75	2.81	1.75	3.00	4
	Easy MD	Infinity Gold	Free	2.00	1.75	2.33	3.00	2.27	1.25	2.00	1
	MD Plan	Diet & Fitness	Free	2.00	3.75	2.33	2.60	2.67	1.50	3.00	2
	MD and Meal Plan	Velapps	Free	2.00	3.25	2.33	2.75	2.58	1.75	3.17	3
	MD	RBJMobileApp	Free	2.40	4.00	3.33	2.50	3.06	2.00	2.00	2
	MD Beginners Plan	Eric Dong	Free	1.80	3.25	2.33	2.60	2.50	1.25		3

Table S1 (continued)

Platform	App Name	Developer	Price (£)	Engagement	Functionality	Aesthetics	Information	Quality mean score	Subjective quality	App-specific quality	Number of BCTs
**Google Play**	MD, the best diet for you?	AcadeMe Hub	Free	3.00	3.75	3.00	3.60	3.34	2.00	2.17	4
	MD plan personalized weight loss	Alebg	Free	3.60	3.75	3.33	2.50	3.30	2.25	2.00	1
	MD 7 Days Meal Plan	Gippy	Free	2.80	3.25	2.33	2.80	2.80	1.75	2.50	3
	Complete MD Cookbook for Beginners	Eric Dong	Free	2.00	3.75	3.00	1.75	2.63	1.50	2.33	1
	MD Plan	Big Caterpillar	Free	3.20	3.50	3.00	3.40	3.28	2.00	2.67	1
	30 Day MD Challenge	Eric Dong	Free	2.00	3.75	2.67	2.80	2.80	2.00	2.33	2
	Mediterranean Recipes: Diet & Meal Planner App	Riafy Technologies	Free	3.60	3.25	3.00	2.67	3.13	2.00	2.50	2
	MD Meal Plan	Health & Fitness Co.	Free	2.00	3.25	3.00	3.00	2.81	1.50	2.50	2
	MD	Healthy Bytes	Free	2.00	3.50	2.33	3.00	2.71	1.50	2.00	2
	850+ Mediterranean Recipes	Startup Media	Free	2.00	3.25	2.67	2.75	2.67	1.50	3.83	2
	MD Recipes	Content Arcade Apps	Free	2.40	3.75	3.33	2.75	3.06	1.75	3.50	2
	The MD	Catholic Prayers	Free	2.20	3.75	2.67	3.40	3.00	1.75	2.67	5
	MD Plan and Recipes	Shopno Apps	Free	1.60	3.50	2.00	2.25	2.34	1.25	3.83	1
	PEP: MD - Food tracker and recipes	Black Bears	Free	3.40	4.00	3.33	3.20	3.48	2.75	3.83	2
	MD Recipes Plan	Adonai Corporation	Free	2.20	3.00	2.67	2.25	2.53	1.50	3.33	2
	MD	Marxis Lozada	Free	1.80	3.75	3.33	2.50	2.85	1.75	2.67	2
	MD Recipes Free	elicoriapps	Free	1.60	3.00	2.33	2.25	2.30	1.25	4.00	2

Table S1 (continued)

Platform	App Name	Developer	Price (£)	Engagement	Functionality	Aesthetics	Information	Quality mean score	Subjective quality	App-specific quality	Number of BCTs
**Google Play**	MD Meal Plan	devcoin	Free	1.60	3.00	1.67	2.50	2.19	1.25	4.00	2
	MD Plan	lahccenappsinc	Free	2.00	3.50	3.00	2.00	2.63	1.50	3.67	2
	MD Meal Plan	AngelicMiho	Free	1.60	2.75	1.33	2.25	1.98	1.25	2.33	2
	MD Meal Plan	RK Unit	Free	1.60	3.25	2.00	2.00	2.21	1.25	3.00	2
	MD Recipes Easy	Noomnim Guide Dev	Free	1.60	3.25	2.33	2.00	2.30	1.25	3.17	2
	MD Recipes Book	Zayn Media	Free	2.80	3.50	2.33	2.50	2.78	1.75	2.33	2
	Authentic MD meal Plan	Matanopp	Free	1.60	3.00	2.33	2.00	2.23	1.25	4.00	2

The quality mean score denotes the average of Engagement, Functionality, Aesthetics and Information scores. Responses of all MARS domains ranged from 1 to 5. Higher scores indicated a higher degree of app quality. Values for the number of BCTs ranged from 0 to 26.
BCTs: Behaviour change techniques; MARS:Mobile Application Rating Scale; MD: Mediterranean diet.
